# A pilot study of a non-invasive oral nitrate stable isotopic method suggests that arginine and citrulline supplementation increases whole-body NO production in Tanzanian children with sickle cell disease

**DOI:** 10.1016/j.niox.2017.12.009

**Published:** 2018-04-01

**Authors:** Alphonce I. Marealle, Mario Siervo, Sara Wassel, Les Bluck, Andrew M. Prentice, Omary Minzi, Philip Sasi, Appolinary Kamuhabwa, Deogratias Soka, Julie Makani, Sharon E. Cox

**Affiliations:** a Muhimbili Wellcome Programme, Muhimbili University of Health and Allied Sciences, Dar es Salaam, Tanzania; b School of Pharmacy, Department of Clinical Pharmacy & Pharmacology, Muhimbili University of Health & Allied Sciences, Dar es Salaam, Tanzania; c Human Nutrition Research Centre, Institute of Cellular Medicine, Newcastle University, Campus for Ageing and Vitality, Newcastle on Tyne, UK; d MRC Human Nutrition Research, Cambridge, UK; e MRC Unit, The Gambia and MRC International Nutrition Group, London School of Hygiene & Tropical Medicine, London, UK; f School of Medicine, Department of Clinical Pharmacology, Muhimbili University of Health & Allied Sciences, Dar es Salaam, Tanzania; g School of Tropical Medicine & Global Health, Nagasaki University, Nagasaki, Japan; h Dept of Population Health, London School of Hygiene & Tropical Medicine, London, UK

## Abstract

**Background:**

Low bioavailability of nitric oxide (NO) is implicated in the pathophysiology of sickle cell disease (SCD). We designed a nested pilot study to be conducted within a clinical trial testing the effects of a daily ready-to-use supplementary food (RUSF) fortified with arginine (Arg) and citrulline (Citr) vs. non-fortified RUSF in children with SCD. The pilot study evaluated 1) the feasibility of a non-invasive stable isotope method to measure whole-body NO production and 2) whether Arg+Citr supplementation was associated with increased whole-body NO production.

**Subjects:**

Twenty-nine children (70% male, 9–11years, weight 16.3–31.3 kg) with SCD.

**Methods:**

Sixteen children received RUSF+Arg/Citr (Arg, 0.2  g/kg/day; Citr, 0.1  g/kg/day) in combination with daily chloroquine (50 mg) and thirteen received the base RUSF in combination with weekly chloroquine (150 mg). Plasma amino acids were assessed using ion-exchange elution (Biochrom-30, Biochrom, UK) and whole-body NO production was measured using a non-invasive stable isotopic method.

**Results:**

The RUSF+Arg/Citr intervention increased plasma arginine (P = .02) and ornithine (P = .003) and decreased the ratio of asymmetric dimethylarginine to arginine (P = .01), compared to the base RUSF. A significant increase in whole-body NO production was observed in the RUSF-Arg/Citr group compared to baseline (weight-adjusted systemic NO synthesis 3.38 ± 2.29 μmol/kg/hr vs 2.35 ± 1.13 μmol/kg/hr, P = .04). No significant changes were detected in the base RUSF group (weight-adjusted systemic NO synthesis 2.64 ± 1.14 μmol/kg/hr vs 2.53 ± 1.12 μmol/kg/hr, P = .80).

**Conclusions:**

The non-invasive stable isotopic method was acceptable and the results provided supporting evidence that Arg/Citr supplementation may increase systemic NO synthesis in children with SCD.

## Highlights

• This method for measuring whole-body NO synthesis is feasible in young children.
• The isotopic decay of the stable isotope tracer demonstrated good linear fit.
• Arginine and citrulline supplementation may increase whole-body NO synthesis.

## 1 Introduction

NO synthase (NOS) converts l-arginine into equimolar amount of NO and citrulline [1]. Citrulline is also a precursor for de novo renal arginine synthesis and, due to a slower hepatic clearance, citrulline supplementation may effectively increase systemic arginine concentrations [2]. A reduced nitric oxide (NO) bioavailability is thought to underlie vascular complications associated with Sickle Cell Disease (SCD) [3]. Arginine is metabolized by the arginase enzyme to ornithine which is a competitive inhibitor of arginine uptake by endothelial cells [4]. Clinical conditions associated with excessive haemolysis, such as SCD and malaria, are characterized by an excessive release of arginase from erythrocytes and consequent reduction of plasma arginine concentrations (i.e., hypo-argininemia) [2,5,6].

Stable isotopic methods used for the measurement of systemic whole-body NO synthesis are mostly based on the oral administration or intravenous infusion of labelled arginine and collection of repeated blood and urine samples [7]. Less invasive methods to measure NO availability are based on the measurement of NO metabolites in plasma (i.e., nitrite, nitrate, cGMP) but the validity and reproducibility of these biomarkers is limited as results may be confounded by factors such as dietary nitrate intake, kidney function or sensitivity of analytical methods [8]. Siervo et al. validated a non-invasive stable isotopic method to provide a quantitative and more accurate estimate of systemic whole-body NO synthesis in humans (Oral Nitrate Test, ONT) [9]. This method employs a very small oral dose of labelled sodium nitrate followed by the collection of repeated saliva samples over a period of 18 h [9].

We conducted a pilot study to determine the feasibility of the ONT isotopic method to measure systemic NO synthesis in a sub-sample of Tanzanian children with SCD enrolled in the second phase of a random-order, double-blind, cross-over clinical trial (NCT01718054). A secondary objective was to determine whether daily supplementation with a ready-to-use-supplementary food (RUSF) fortified with arginine and citrulline [RUSF+Arg/Cit] increased systemic NO production compared to children receiving RUSF without Arg/Cit fortification.

## 2 Methods

**Fig. 1:**
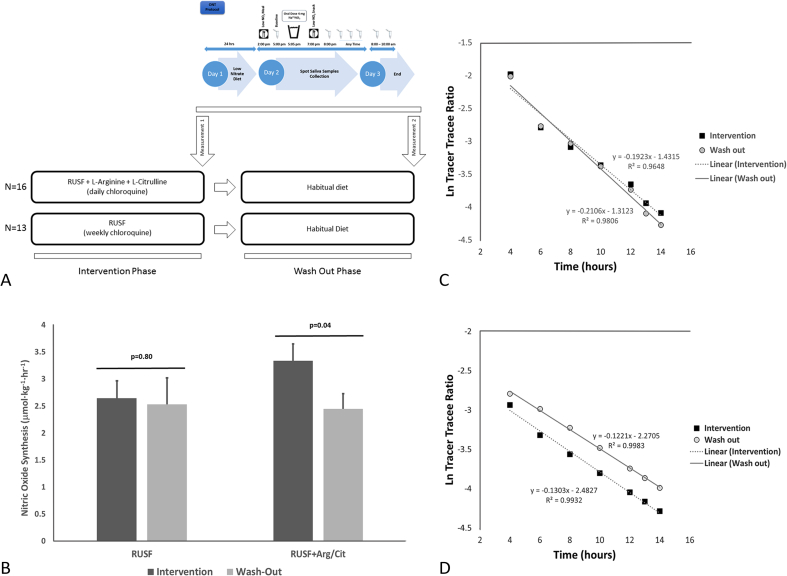
**A**: Study design. Figure summarises the oral nitrate test (ONT) method used to measure nitric oxide synthesis and the time points of sample collection from children in the 2nd phase of the V-FIT cross-over trial. **B** Differences in whole body NO synthesis measured using the ONT between supplemented (RUSF+Arg/Citr in combination with daily chloroquine [50 mg]) and non-supplemented arms (base RUSF in combination with weekly chloroquine [150 mg]) during the intervention and wash out period. Data presented as mean±SEMs and P-values from paired Wilcoxan tests for non-parametric data. **C and D** shows the time-course of the isotopic disappearance of the tracer, expressed as tracer-tracee ratio, followed for 14 h after the administration of an oral dose of labelled sodium nitrate (Na^15^NO_3_). The mean values of the tracer-tracee ratio for the saliva kinetic curves are reported on a semi-logarithmic scale and shown for the base RUSF (Fig. 1C) and RUSF+Arg/Citr (Fig. 1D) arms.

Twenty-nine children (70% male, 9–11 years, weight 16.3–31.3 kg) with SCD were enrolled in this study during the second intervention phase of the cross-over clinical trial. The study protocol was approved by the London School of Hygiene and Tropical Medicine and Muhimbili University of Health and Allied Sciences institutional review boards. All parents/guardians provided informed consent and all children assented. Sixteen children received RUSF+Arg/Cit (arginine, 0.2  g/kg/day; citrulline, 0.1  g/kg/day) in combination with daily chloroquine (50 mg) and thirteen received the base RUSF in combination with weekly chloroquine (150 mg). Both RUSF formulations provided 500 kcal, 1 RDA of vitamins and minerals & 1 mg of folate (Nutriset, France). The ONT method measures the decay of an oral dose of labelled sodium nitrate in serial saliva samples [9]. Briefly, children received a controlled low nitrate meal and a baseline saliva sample was collected 4 h after the meal. This was followed by the ingestion of an oral dose of 4 mg of Na^15^NO_3_ (^15^N, 98% +, Cambridge Isotope Laboratories, Inc., Andover, MA, USA) in 100 mL of nitrate-free water. The remaining samples were collected at 6, 7, 8, 17 and 18 h post-meal. Measurements of nitrate enrichments in saliva were conducted using Gas Chromatography Mass Spectrometry as described by Siervo et al. [9]. The isotopic decay of the oral dose of labelled nitrate was defined by an exponential function for a single compartment which was employed to calculate the rate of whole-body NO production [9]. Amino acid concentrations were measured in frozen lithium heparin plasma samples (Biochrom-30 amino acid analyzer (Biochrom, UK)) at the end of each intervention period and at baseline entry into the main trial. More detailed descriptions of the ONT and amino acid analyses are provided in the online supplementary material. Whole-body NO production using the ONT method was assessed at the end of each intervention phase after a mean duration of approximately 10.7 weeks. NO production was assessed again after a washout period of approximately 8 weeks (range 5–12 weeks) in both intervention groups (Fig. 1A). The sample size was calculated using data of NO production measured by the ONT method in obese subjects with and without metabolic syndrome (3). A sample size of nine subjects per group was estimated to have 90% power to detect a difference in whole-body NO production using the ONT method similar to that observed between obese adults with (0.21 ± 0.13μmol/hr/kg) and without (0.49 ± 0.22 μmol/h/kg) metabolic syndrome [9].

Mann-Whitney *U* test was used to assess significant differences in NO production between the RUSF+Arg/Cit and base RUSF intervention groups. The paired Wilcoxon test was used to assess significant changes in NO production between supplementation and wash-out periods within the same intervention group. Analyses were performed using SPSS 22 for Windows (IBM, USA).

## 3 Results

Age and sex were not associated with NO production (data not shown). The RUSF+Arg/Cit intervention significantly increased plasma arginine, ornithine, non-significantly increased citrulline and significantly decreased the ratio of asymmetric dimethylarginine (ADMA) to arginine compared to the base RUSF intervention. The global arginine bioavailability ratio (GABR; arginine/ornithine+citrulline) and the ratio of arginine to ornithine were not statistically different between the two interventions (Table 1).

**Table 1:** Age, sex, weight and plasma amino acid concentrations involved in nitric oxide synthesis in the two groups of children during intervention either with RUSF+ARG/CIT or standard RUSF.

	RUSF+ARG/CITR	RUSF	P^a^
N	16	13	
Age (years)	11.06 ± 1.23	11.30 ± 1.25	0.68
Gender (M/F)	13/3	7/6	0.11
Weight (kg)	24.61 ± 3.52	25.18 ± 3.43	0.75
Arginine (nmol/mL)	130.16 ± 115.65	62.11 ± 15.58	0.02
Ornithine (nmol/mL)	101.05 ± 71.00	52.33 ± 12.81	0.003
Citrulline (nmol/mL)	48.19 ± 62.66	22.18 ± 6.02	0.07
ADMA (nmol/mL)	0.95 ± 0.25	0.96 ± 0.24	0.84
Arginine/Ornithine Ratio	1.22 ± 0.35	1.20 ± 0.23	0.89
ADMA/Arginine Ratio	0.011 ± 0.007	0.016 ± 0.006	0.01
GABR	0.86 ± 0.23	0.84 ± 0.17	0.49

Data are presented as mean±SD. N = number of subjects; M = Male; F = Female. ADMA = asymmetric dimethylarginine. RUSF = ready-to-use supplementary food. ARG = arginine; CITR = citrulline; GABR = Global arginine bioavailability ratio. Mann-Whitney test was used to compare the two intervention groups.

a P value is for comparison of the two intervention groups.

Baseline NO synthesis in children with SCD was 2.32 ± 0.97 μmol/kg/h. The difference in NO synthesis between the treatment and washout periods was significant in the RUSF-Arg/Citr group (P = .04) but not in the RUSF group (P = .80) (Fig. 1B**).** The isotopic decay of the stable isotope tracer was characterized by a good linear fit in both groups; the effect of the interventions on systemic NO production is described by the differences in isotopic decays in the RUSF (Fig. 1C) and RUSF+ARG/CIT (Fig. 1D) groups. However, the difference in NO synthesis between the RUSF-Arg/Citr and RUSF groups at the end of the intervention was not significant (P = .31). NO synthesis was not correlated with citrulline, arginine and ADMA concentrations, and ADMA to arginine ratio (data not shown).

## 4 Discussion

The acceptability of the ONT method in children with SCD was good. This is the first study to report measurements of systemic NO production in non-hospitalized children with SCD using a non-invasive stable isotopic method, which may promote further research into the role of NO in SCD and other conditions characterized by impaired NO regulation. Baseline NO synthesis rates in children with SCD (2.32 ± 0.97 μmol/kg/h) were greater than healthy adults (0.63 ± 0.20 μmol/kg/h) and obese subjects with metabolic syndrome (0.21 ± 0.13 μmol/kg/h) using the ONT method [9]. The NO synthesis rates observed in our study were also greater than rates measured in hospitalized children with uncomplicated malaria using an invasive method requiring an intravenous administration of labelled ^15^N-arginine (0.83 ± 0.11 μmol/kg/h; range 0.3–2.65 μmol/kg/h) and collection of total urine output over a period of 48 h [10]. A lower systemic NO synthesis was also measured in ten critically ill children with sepsis (NO synthesis rate = 1.58 ± 0.7 μmol/kg/h) and differences with our study could be related to disease pathogenesis and/or characteristics of the stable isotope methods [11]. However, two studies found similar (2.4 ± 0.6 μmol/kg/h) [12] or higher (4.3 ± 2.7 μmol/kg/h) [13] NO synthesis rates in adult patients with cirrhosis and end stage renal disease, respectively, using isotopic methods based on the intravenous administration of labelled arginine. There is currently limited information on the NO synthesis rates in pediatric populations. The only study to evaluate the efficiency of the enzymatic NO synthetic pathway in children was conducted by Forte et al. [14]. The study used oral labelled arginine in 17 healthy children aged 4–16 years old to measure the percentage of L-[^15^N]_2_-guanidino arginine dose directed to NO synthesis but it did not report rates of whole-body NO synthesis. The study found that the proportion of labelled arginine directed to NO synthesis was 0.22% and also observed that the percent rate of incorporation was inversely associated with age (r = −0.53, p < .05); we did not observe a similar association between age and NO synthesis which could be due to the narrower age range of our population.

A previous study has shown that oral supplementation with arginine and citrulline increases NO synthesis in patients with SCD [15,16] and improves NO-mediated endothelial function in adults with impaired vascular health [17]. The higher NO synthesis observed in our study suggest that NO synthesis in children with SCD may not be affected by a reduced availability of arginine and the elevated rate of whole-body NO synthesis may be the result of compensatory responses to an increased rate of NO degradation [18,19].

However, this study is characterized by several limitations which need to be taken into account in the interpretation of the results. Systemic NO production measured by the ONT method reflects the contribution of both enzymatic and non-enzymatic NO synthetic pathways; however, the controlled low nitrate diet followed by the children as part of the ONT protocol was implemented to reduce the contribution of the non-enzymatic pathway [20]. In addition, there is currently no evidence on the effects of arginine, citrulline and nitrate supplementation on the efficiency of bacterial and tissue nitrate reductase activity and therefore the impact of these nutrients and drug on the rate of conversion of nitrate into NO via the non-enzymatic synthetic pathways is undetermined. It is also important to highlight that an increase in NO synthesis and overall bio-availability does not necessarily correspond to an increased NO-mediated activation of cellular mechanisms. It is possible that the observed increase in systemic NO production in the RUSF-Arg/Cit group was the result of the higher dose of chloroquine in this group, rather than from a greater supply of arginine from the fortificants, which may have reduced arginase activity [21] and/or inflammation [22,23] and result in greater NO bioavailability in children with SCD [24]. However, there was no difference in the arginine:ornithine ratio between the two groups and therefore a different degree of inhibition of arginase activity by chloroquine administration seems unlikely. In addition, weekly doses of chloroquine in the RUSF group did not have an effect on NO synthesis. While we found a decrease in the ADMA to arginine ratio between the two intervention groups, the within-intervention changes of the ratio were not different between the two groups, which, if added to a lack of significant correlation of the ADMA to arginine ratio with whole-body NO production, seems to suggest that ADMA was not linked to the increase in NO synthesis. Finally, a comparison of systemic NO production rates measured in children with SCD and age-matched healthy controls should be investigated in future studies.

The application of the non-invasive isotopic method for the measurement of systemic NO production is feasible in this population and our results suggest that supplementation with arginine and citrulline may increase NO synthesis in children with SCD, although it is unclear by what mechanism.

## 5 Trial registration

V-FIT is registered on clinicaltrials.gov with ID number NCT01718054.
