# Supplementary material for: A pilot study of a non-invasive oral nitrate stable isotopic method suggests that arginine and citrulline supplementation increases whole-body NO production in Tanzanian children with sickle cell disease
Source: Nitric Oxide. 2018 Apr 1;74:19–22. doi: 10.1016/j.niox.2017.12.009 (PMC5832986; doi:10.1016/j.niox.2017.12.009)
Supplement: Supplementary materials [file mmc1.docx]

**SUPPLEMENTARY MATERIAL**

Oral Nitrate Test (ONT)

The participants were required to arrive at the V-FIT research clinic at 1000 h having not fasted and stayed overnight at a full time staffed facility (Tumaini Hospital private rooms). At 1300 h participants were served with a low nitrate standard meal (rice, chicken, and fruits) which was to be consumed within 30 minutes. The meals were formulated to have an energy content of ~1400 kcal and provide a NO3- intake <5 mg/meal (estimation was based on UK foods). Each participant was encouraged to eat as much food as they could but they were not necessarily required to eat all the food provided. After the meal, participants were instructed not to eat and to drink only the low nitrate water provided (Masafi 0.8mg nitrates) until 1900 h when they were provided with a low nitrate snack which was to be consumed within 30 minutes. Thereafter the participants were instructed again and monitored not to eat and to drink only the low nitrate water for a total fasting time of 14 h until the next day morning at 0900 h. At 1750 h pre-dose (baseline) induced saliva samples were collected. After the collection of the baseline samples, immediately participants were given an oral dose of 4mg of Na^15^NO_3_ (Cambridge Laboratories Inc) in 100ml of distilled water at around 1800 h. The next saliva samples were collected at 2030 h followed by three more samples which were collected at 2200 h, 2300 h and 0000 h. The last two samples were collected the next day morning at 0800 h and 0900 h.

Saliva sample processing and storage

Stimulated saliva samples were collected using cotton wool method in 1.5 ml eppendorf tubes containing 3.7µl of 5M sodium hydroxide. To stimulate saliva production, each participant was asked to chew a small ball of cotton wool for about 2 minutes. The cotton ball was then inserted into a barrel of a 30ml syringe and the plunger was used to squeeze the saliva into the 1.5mL vials. The aliquots were immediately stored at in a cool box until when all samples were collected from each participant and then transferred to -20.0C freezer at Muhimbili Wellcome Programme (MWP). Two aliquots were collected for each sample making a total of 14 saliva samples from each participant in each phase. Therefore 14 samples were collected during the intervention period and other 14 samples were collected during the washout period. The samples collected during the wash out period to allow an estimation of baseline NO oxide production rate. Salivary nitrate enrichments and concentrations are stable at room temperature for at least 48 hours. After storage at -20C, no changes in concentrations and enrichments were observed over a period of 8 weeks^1^.

Nitrate measurements and modelling of results

Enrichment: All saliva samples were derivatised by nucleophilic substitution of mesitylene with trifluoroacetic acid anhydride (TFAA) as the catalyst to give a single product, nitromesitlylene (1, 3, 5-trimethyl nitrobenzene). Gas chromatography Mass Spectrometry was used to determine the level of enrichment. The full description of the method is described elsewhere along with GC-MS conditions. All analyses were performed in duplicate in selected ion monitoring (SIM) of the molecular ion (MO) at m/z 165 and the M+1 ion at m/z 166 representing the unlabelled and labelled nitromesitlylene respectively^2^.

The oral dose of labelled nitrate is rapidly assimilated into plasma (complete absorption in under 2 h), which, on the timescale of the disposal kinetics, can be regarded as a bolus dose. Linear interpolation was used to estimate the 2-h changes in concentrations and tracer-to-tracee ratio (TTR) during the fasting period.

The tracer to tracee ratio (TTR) of the solutions were calculated as the ratio of the labeled tracer/unlabeled tracee:

TTR = (^15^N/^14^N)*100

The level of isotopic enrichment for each sample was calculated as the difference between the tracer-tracee ratio (TTR) of the sample and the measured isotopic background abundance of nitromesitylene. The Atom Percent Excess (APE) was calculated as follows:

APE = TTR/(TTR+1) *100

The range of isotopic enrichment reached 2-hr post dose varied between 12.9 and 25.2% with an average enrichment of 17.3±2.8%. The isotopic decay in saliva of an oral dose of labelled nitrate was described by an exponential function for single compartment. Data was described using a semi-logarithmic plot and the slope and intercept of the regression line was used to derive the rate of NO synthesis. The intercept of the model provides an estimate of the nitrate pool at time 0 whereas the slope represents the rate of unlabelled nitrate, derived from endogenous NO synthesis, enriching the nitrate pool over time^1^. It should be noted that although the concentration of nitrate in saliva and plasma are not equal the TTR is expected to be the same. Finally, nitrate represents the stable end product (half-life: ~6-8 hours) of NO metabolism and, therefore, its generation reflects the synthetic rate of endogenous NO when exogenous nitrate sources have been controlled. Nitrite is short lived (half-life: ~5-10 minutes) and within minutes is converted into nitrate as part of the NO-nitrite-nitrate catabolic process. The assessment of the isotopic decay of unlabelled and labelled nitrate over 18 hours means that nitrite is converted to nitrate, thus avoiding any potential interference on the assessment of NO production. The model is based on the assumption that salivary nitrate is a proxy measure of the plasma nitrate pool. Previous studies have demonstrated a significant association between salivary and plasma nitrate in (r=0.36, p=0.001)^3^; and (r=0.55, p=0.04)^4^ but no significant association was found between salivary and plasma nitrite concentrations (r=0.002, p=0.96)^3^.

Amino acid analysis

Lithium heparin plasma samples were processed and, archived at -80 within 2 hours of blood collection and later analysed using a Biochrom 30 amino acid analyser. This employs ion exchange chromatography with stepwise elution gradient and spectrophotometric detection at 570 nm and 440 nm following ninhydrin post-column derivatization.

References

1. Mario Siervo. Measurement of in Vivo Nitric Oxide Production using Stable Isotopes. University of Cambridge, PhD Thesis, 2012.
2. Jackson SJ, Siervo M, Persson E, McKenna LM, Bluck LJ. A novel derivative for the assessment of urinary and salivary nitrate using gas chromatography/mass spectrometry. Rapid Commun Mass Spectrom. 2008 Dec;22(24):4158-64
3. Sukuroglu E, Güncü GN, Kilinc K, Caglayan F. Using Salivary Nitrite and Nitrate Levels as a Biomarker for Drug-Induced Gingival Overgrowth. Front Cell Infect Microbiol. 2015 Dec 1;5:87.
4. Clodfelter WH, Basu S, Bolden C, Dos Santos PC, King SB, Kim-Shapiro DB. The relationship between plasma and salivary NOx. Nitric Oxide. 2015 May 1;47:85-90
